# Separation and Identification of an Antimicrobial Substance from *Schisandra chinensis* Extract against *Streptococcus mutans* KCCM 40105 Strain

**DOI:** 10.3390/molecules28083417

**Published:** 2023-04-13

**Authors:** Jae-Hee Jeong, Su-Hwan Kim, Chang-Ki Huh

**Affiliations:** 1 Department of Food Science and Technology, Sunchon National University, Suncheon 57922, Republic of Korea; 2 Research Institute of Food Industry, Sunchon National University, Suncheon 57922, Republic of Korea

**Keywords:** *Schizandra chinesis*, *Streptococcus mutans*, antimicrobial substance, tartaric acid

## Abstract

This study aimed to isolate and identify antibacterial compounds from *Schisandra chinensis* (*S. chinensis*) that are effective against the Streptococcus mutans KCCM 40105 strain. First, *S. chinensis* was extracted using varying concentrations of ethanol, and the resulting antibacterial activity was evaluated. The 30% ethanol extract of *S. chinensis* showed high activity. The fractionation and antibacterial activity of a 30% ethanol extract from *S. chinensis* were examined using five different solvents. Upon investigation of the antibacterial activity of the solvent fraction, the water and butanol fractions showed high activity, and no significant difference was found. Therefore, the butanol fraction was chosen for material exploration using silica gel column chromatography. A total of 24 fractions were obtained from the butanol portion using silica gel chromatography. The fraction with the highest antibacterial activity was Fr 7. From Fr 7, thirty-three sub-fractions were isolated, and sub-fraction 17 showed the highest level of antibacterial activity. A total of five peaks were obtained through the pure separation of sub-fraction 17 using HPLC. Peak 2 was identified as a substance exhibiting a high level of antibacterial activity. Based on the results of UV spectrometry, 13C-NMR, 1H-NMR, LC-MS, and HPLC analyses, the compound corresponding to peak number 2 was identified as tartaric acid.

## 1. Introduction

*Streptococcus mutans* (*S. mutans*) is a major etiological agent of dental caries, an infectious disease caused by oral bacteria capable of colonizing the surface of teeth and producing organic acids such as lactic acid through the metabolism of glucose. In addition, it has the ability to survive in acidic mediums and accumulate intracellular polysaccharides from the fermentation of glucose and other carbohydrates. This process causes a decrease in oral pH, inducing loss of calcium from the surfaces of teeth and demineralization, often resulting in halitosis and stomatitis caused by tooth decay [1,2]. Inhibiting adhesion to the tooth surface, colonization in the oral cavity, and synthesis of insoluble glucans by *S. mutans* is important for the prevention of dental caries [3]. Thus, there is an increasing need for the development of natural substances with antibacterial activity against causative pathogens and periodontitis- or halitosis-causing agents, which are also non-toxic to the human body. The study of natural extracts from commonly utilized food and medicines that are relatively safe for humans has been ongoing in recent years [4].

*Schisandra chinensis* (*S. chinensis*), a member of the Magnoliaceae family, native to East Asia, including Korea, China, and Japan, is a widely utilized medicinal and edible plant [5]. Antioxidant and antibacterial effects of *Schisandra chinensis* against air pollution and fine dust pollution have been reported; thus, this plant has been widely utilized in different fields, including medicine, health food, cosmetics, beauty care products, natural color, and others [6,7].

The fruit of *Schisandra chinensis* is known as ‘Omija’ in Korea because of its unique taste featuring five flavors: sour, sweet, spicy, bitter, and salty [8]. This fruit has been identified as an alkaline food containing various organic acids, including citric acid, malic acid, succinic acid, fumaric acid, and tartaric acid, minerals including K, P, Ca, Mg, Fe, and Mn, and fatty acids including linoleic acid, oleic acid, palmitic acid, and stearic acid [9]. In addition, the fruit contains functional substances, including more than 40 different lignan compounds such as schisandrin A-C, schisandrol, deoxyschizandrin, gomisin A-H, gomisin J-K1, K2, K3, gomisin N, wuwerizilctone acid, and others [10,11,12]. Physiological functions related to *S. chinensis* include reported blood pressure-lowering effects, cholesterol-lowering effects, antioxidant effects, antimicrobial activity, anti-inflammatory effects, and anticancer effects [13]. In addition, medicinal actions include central inhibitory action such as sedative, antitussive, and antipyretic effects, the inhibitory effect of liver injury, antidotal effect against alcohol, and others. Studies investigating lignan compounds, the major pharmacologically functional component, have been primarily reported [14,15,16]. Previous studies have reported on the antibacterial effect of seed and fruit extract of *S. chinensis* [17,18]. Earlier studies also focused predominantly on determining the antibacterial effects on foodborne pathogens, resident skin flora, and others [19]. However, studies on antimicrobial activity against oral bacteria (*S. mutans*) have rarely been reported. Additional studies and needed to confirm the activity against oral bacteria (*S. mutans*). Furthermore, this study aimed to establish a foundation for application to various products that can effectively remove various microorganisms that can remain in the oral cavity and exhibit antibacterial effects. Therefore, in this study utilizing a natural antibacterial substance against *S. mutans* KCCM 40105 strain, a natural substance from the butanol fraction of *S. chinensis* 30% ethanol extract was isolated using silica gel column chromatography and HPLC, and the active antibacterial substance was confirmed by identification using UV spectrum, HPLC, LC-MS, and nuclear magnetic resonance (NMR).

## 2. Results and Discussion

### 2.1. Antimicrobial Activity of S. chinensis Extract According to Ethanol Concentration

**Figure 1:**
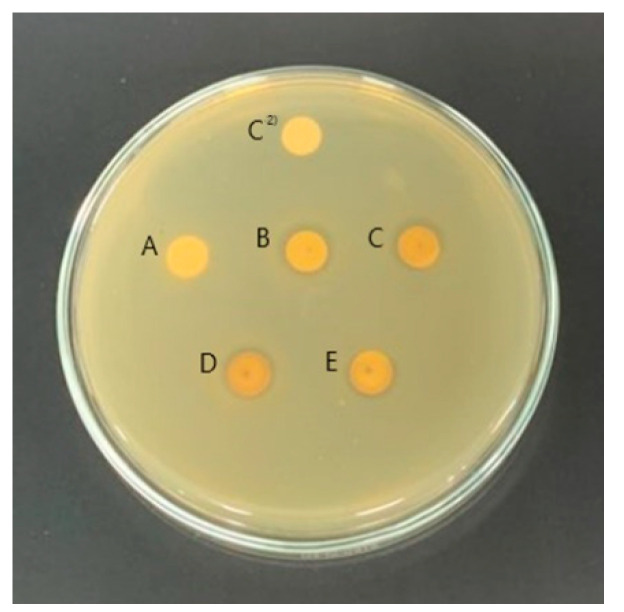
Antibacterial activity according to ethanol concentration of *Schisandra chinensis* against *Streptococcus mutans* KCCM 40105 strain. C^2^, control; A, water extract; B, 30% ethanol extract; C, 50% ethanol extract; D, 70% ethanol extract; E, 94.5% ethanol extract.

The results of measuring the antimicrobial activity of *S. chinensis* extract, according to ethanol concentration, showed activity at all concentrations. The antimicrobial activity of the water extract was 12.92 mm, while the 30%, 50%, 70%, and 94.5% ethanol concentrations showed activities of 14.49 mm, 14.67 mm, 13.36 mm, and 9.68 mm, respectively (Figure 1). The results of the antibacterial activity measurement showed that the activity increased up to a 50% ethanol concentration. However, the activity decreased in extracts with higher concentrations of ethanol, greater than 50%. Therefore, in order to apply *S. chinensis* extract to various products, a low concentration of ethanol solvent is more suitable for industrialization than a high concentration of ethanol solvent. Therefore, the ethanol concentration for *S. chinensis* extraction was selected as 30%.

### 2.2. Antibacterial Activity according to the Solvent Fractionation of the 30% Ethanol Extract of S. chinensis

**Figure 2:**
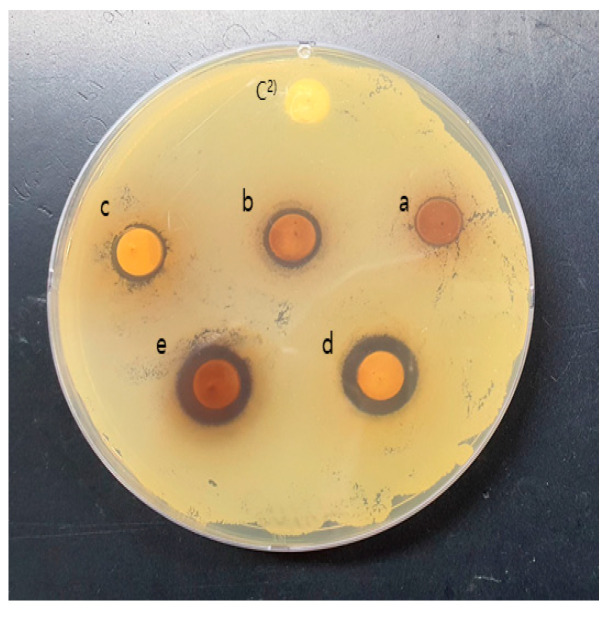
Antibacterial activity according to various solvent fractionation of *Schisandra chinensis* 30% ethanol extract against *S. mutans* KCCM 40105 strain. C^2^, Control; a, Hexane fraction; b, Chloroform fraction; c, Ethyl acetate fraction; d, n-Butanol fraction; e, Water fraction.

Solvent fractionation was conducted to search for antimicrobial substances in the 30% ethanol extract of *S. chinensis*. The solvents used were hexane, chloroform, ethyl acetate, butanol, and water. The inhibitory activities of five different solvent-extracted fractions were 13.23 mm in the n-butanol layer, 13.28 mm in the water layer, 10.99 mm in the chloroform layer, 10.46 mm in the ethyl acetate layer, and 8.52 mm in the hexane layer (Figure 2). As a result of the antibacterial activity of the solvent fraction, the water and butanol fractions showed high activity, but no significant difference was found. Jung et al. [20] measured the antibacterial activity of the methanol extract of *S. chinensis* seeds against five bacteria (*L. plantarum, B. subtilis, S. aureus, S. typhimurium, E. coli*), according to the solvent fraction, and reported that the butanol fraction showed high activity, which is consistent with the findings of this study. However, Lee et al. [21] reported that the ethyl acetate fraction showed high antibacterial activity as a result of the solvent fraction of the methanol extract of *S. chinensis*, showing a difference from the results of this study. This seems to be attributable to the difference in the part used and the extraction solvent, as well as the physiological function of the microorganisms. Therefore, the butanol fraction was selected for material search through silica gel column chromatography.

### 2.3. Separation and Verification by Silica Gel Column Chromatography

**Figure 3:**
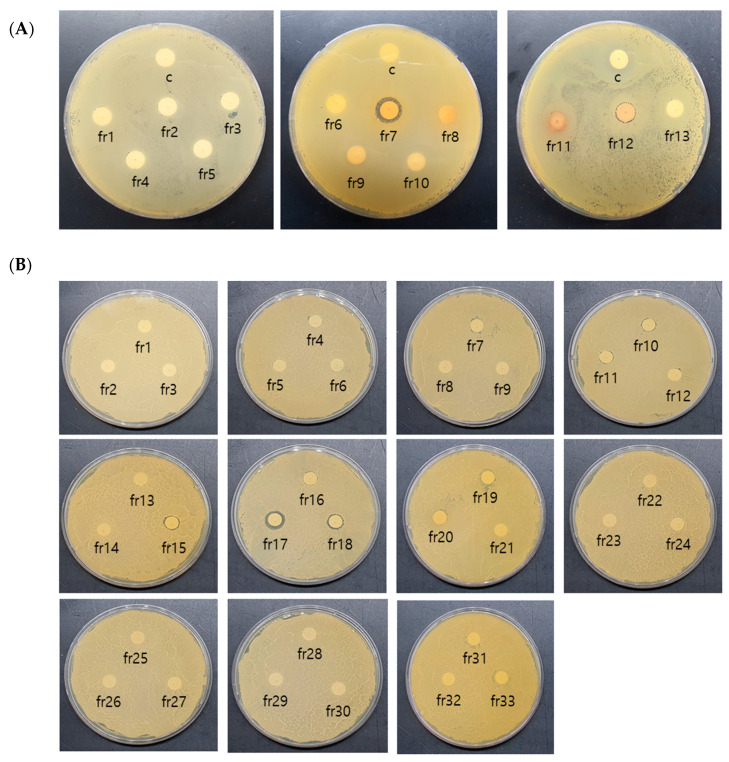
Antibacterial activity of fractions by silica gel column chromatography. (**A**) Antibacterial activity of 1st silica gel column chromatography fractions against *Streptococcus mutans* KCCM 40105 strain., (**B**) Antibacterial activity of 2nd silica gel column chromatography fractions against *Streptococcus mutans* KCCM 40105 strain.

Among the solvent fractions obtained from 30% ethanol extract of powdered *S. chinensis* that exhibited different antimicrobial activities according to the ethanol concentration, a high level of antimicrobial activity was detected in the butanol fraction. Silica-gel column chromatography was performed consecutively for the isolation and identification of active antimicrobial substances in the butanol fraction of *S. chinensis*. A total of 24 sub-fractions were obtained by primary silica gel column chromatography. Figure 3A shows the antimicrobial activities of 13 fractions determined using a spectrophotometer in wavelength scan mode. In the evaluation of 13 fractions, the highest level of inhibitory activity was detected in Fr-7 at 15.27 mm. Secondary silica gel column chromatography was subsequently performed for the separation of Fr-7 fractions.

Secondary silica gel column chromatography was performed in order to isolate the active substance of the Fr-7 fractions, and 33 sub-fractions were collected. The results of the measurement of antimicrobial activities of 33 sub-fractions are shown in Figure 3B. Fractions found to exhibit antimicrobial activity included Fr-15 (9.24 mm), Fr-16 (9.13 mm), Fr-17 (12.81 mm), and Fr-18 (9.91 mm). The strongest antimicrobial effect was observed in Fr-17. HPLC was performed for pure isolation of Fr-17, which exhibited the strongest activity.

### 2.4. Antimicrobial Activity of Pure Isolates by HPLC

**Figure 4:**
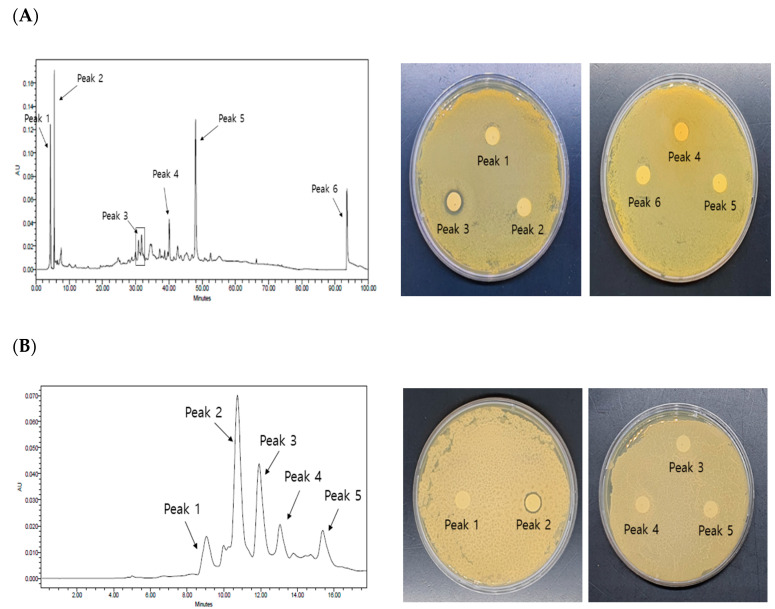
Antibacterial activity of pure isolates by HPLC. (**A**) Antibacterial activity of 1st HPLC pure isolates against *Streptococcus mutans* KCCM 40105 strain. (**B**) Antibacterial activity of 2nd HPLC pure isolates against *Streptococcus mutans* KCCM 40105 strain.

In the process of separation of antimicrobial agents from *S. chinensis* against the *S. mutans* strain, fractionation was performed according to the following steps: drying *S. chinensis* extract powder → 30% ethanol extraction (extraction at different ethanol concentrations) → butanol fraction (fractionation by the solvent) → Fr-7 fraction (primary silica gel column chromatography) → Fr-17 fraction (secondary silica-gel column chromatography). Next, pure isolation of the Fr-17 fraction was performed using HPLC. The results of primary pure isolation are shown in Figure 4A. A total of six peaks were identified, and the third peak exhibited the strongest antibacterial activity.

A chromatogram for the secondary pure isolate obtained by HPLC and the results of the assessment of antimicrobial activity are shown in Figure 4B. In the analysis of the second peak exhibiting antimicrobial activity in primary HPLC, five peaks were identified; secondary pure isolation was performed using HPLC in the range of 320 nm and 254 nm. The results of the analysis indicated five peaks at a wavelength of 254 nm, and antimicrobial activity was detected in the second peak.

### 2.5. Analysis of UV Spectrum

**Figure 5:**
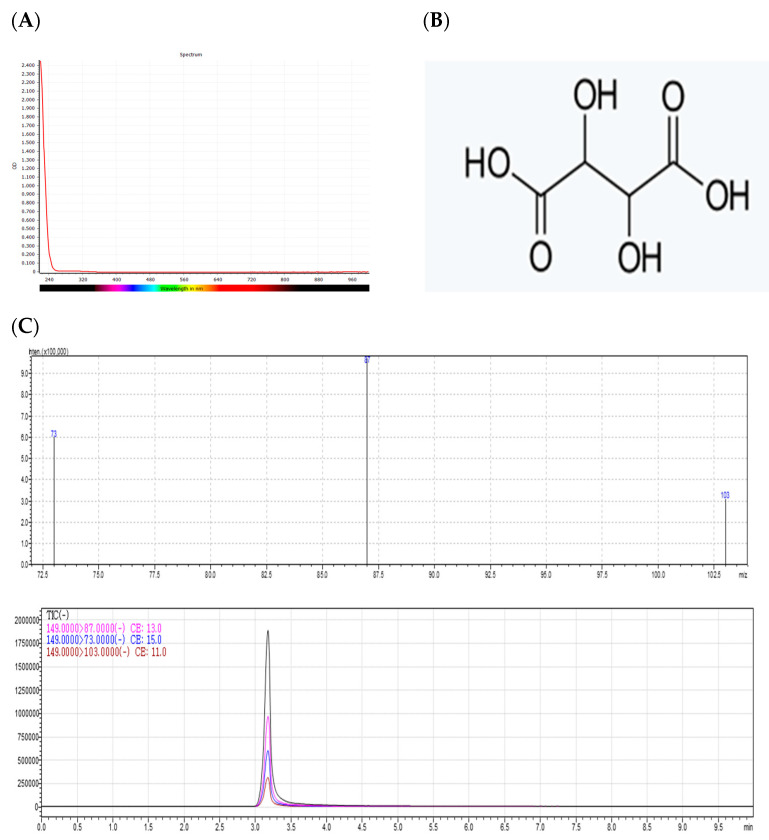

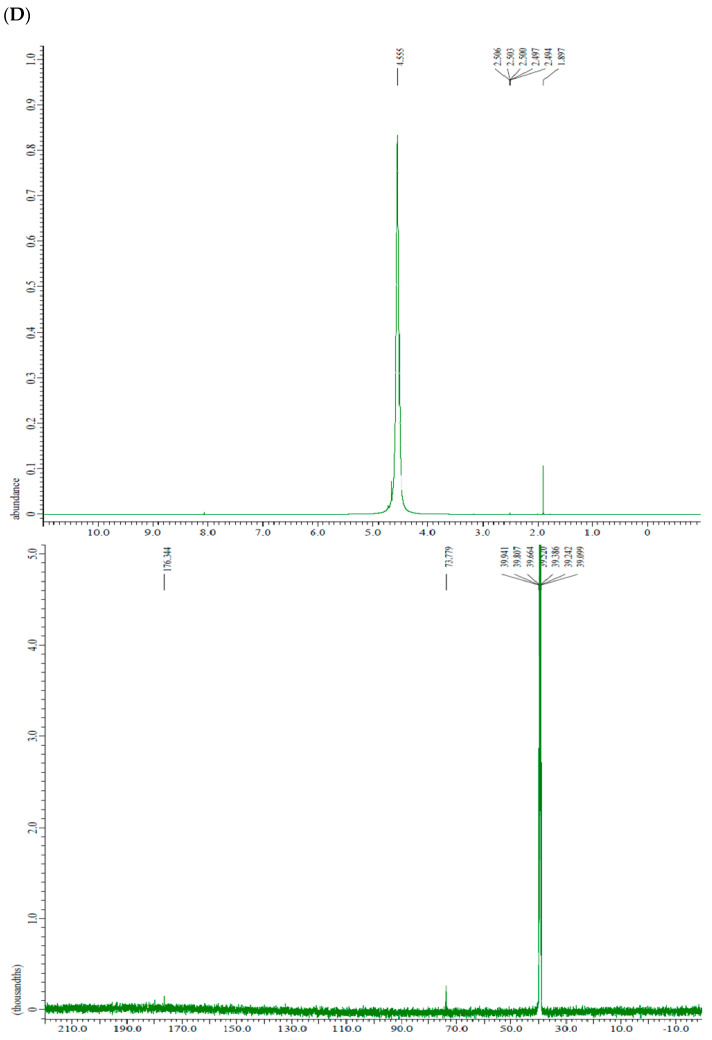
Identification of the *Schizandra chinensis* fraction with confirmed antibacterial activity against *Streptococcus mutans* KCCM 40105 strain. (**A**) UV spectrum of active antimicrobial substances., (**B**) Chemical structures of active antimicrobial substances., (**C**) LC-MS chromatogram and molecular weight of active antimicrobial substances., (**D**) 1H-NMR and 13C-NMR chromatogram of active antimicrobial substances.

The antimicrobial substance in *S. chinensis* against the *S. mutans* strain identified in peak no. 2, which exhibited antimicrobial activity in the secondary process of pure isolation using HPLC, was dissolved in methanol, and measurement of absorbance in the range of 210 to 1000 nm was performed using a microplate reader (SPECTROstarNano, BMG Labtech, Ortenberg, Germany). The results of spectral scanning are shown in Figure 5A. An absorbance peak ranging between 240 and 320 nm was observed for the antimicrobial substance; this compound is believed to be the combination of a conjugated chromophore and an auxochrome.

### 2.6. Molecular Weight Determination by LC-MS

Following the assessment of solvent-partitioned fractions of *S. chinensis* 30% ethanol extracts, silica gel column chromatography was performed for the separation of the butanol fraction that exhibited the strongest antimicrobial activity. Pure isolation of the Fr-17 fraction was performed using HPLC, and an assessment of the molecular weight of the peak no. 2 substance was performed using LC-MS. The results are shown in Figure 5C. The results of the analysis demonstrated the spectral purity of the peak, and LRESIMS m/s was estimated as [H]^+^ 149.0 in negative mode.

### 2.7. Structure Determination by NMR Spectroscopy

The results of NMR spectroscopy to determine the molecular structure of the active substance in Fr-17 peak 2 obtained from the antimicrobial substances of *S. chinensis* are shown in Figure 5D. The chemical structures of fractions identified by high-magnetic-field ^1^H-NMR and ^13^C-NMR spectra analysis included ^1^H-NMR (DMSO, 600 MHz); 4.562 (1H, S), ^13^C-NMR (DMSO, 600 MHz); 73.779 (C1,C2), 176.344 (C3,C4). Based on the Spectral Database System [22,23,24], it was predicted that the substance was tartaric acid.

### 2.8. Material Determination by HPLC

**Figure 6:**
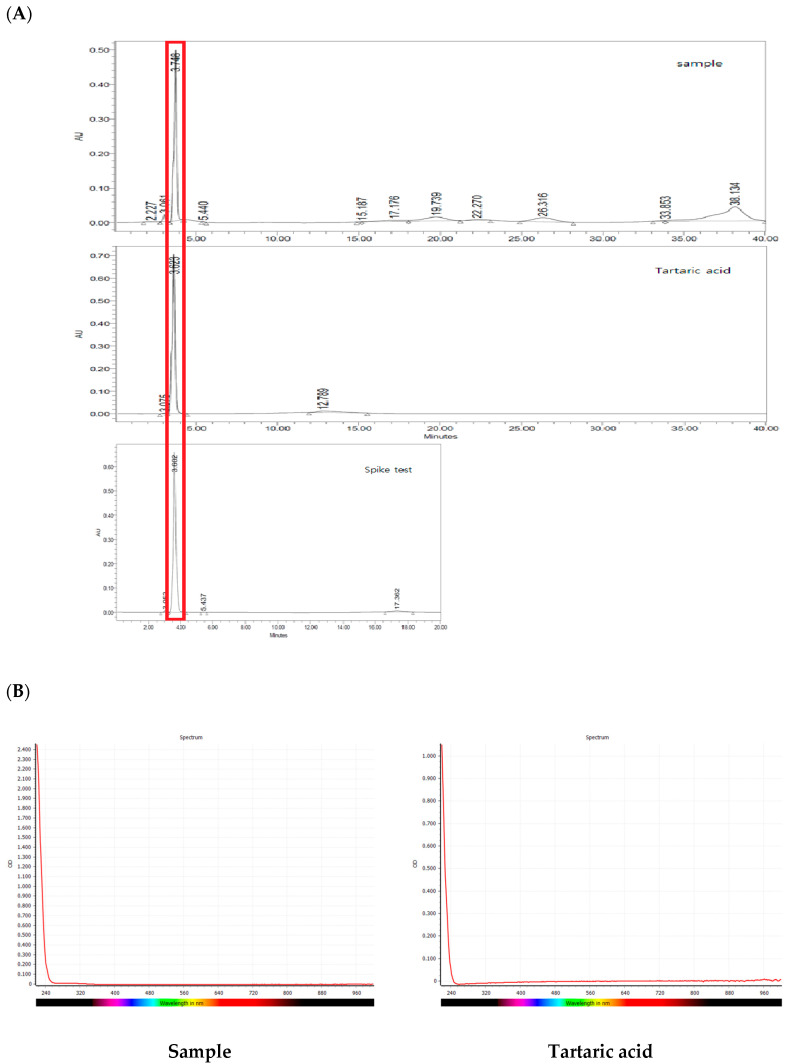
Comparison of HPLC chromatograms and UV spectrum of active antibacterial fractions of *Schizandra chinensis* and tartaric acid standards. (**A**) Comparison of HPLC chromatograms. (**B**) Comparison of UV spectrum. Sample; Active antibacterial substances of *Schizandra chinensis.* Tartaric acid; Standard, Spike test; Active antibacterial substances of *Schizandra chinensis* + tartaric acid standard.

Structure determination using UV spectrum, LC-MS, and NMR spectroscopy resulted in the identification of the active substance in *S. chinensis* Fr-17-peak 2 as tartaric acid. A comparison of chromatograms of the reference materials of tartaric acid and the antimicrobial agent of *S. chinensis* was performed. The results are shown in Figure 6A. The retention time of the antimicrobial substance from *S. chinensis* Fr-17-peak 3 was 3.7483 min. A similar retention time of 3.6923 min was observed for tartaric acid. Using the standard method for the addition of reference materials, the chromatogram of the reference materials for tartaric acid matched with that of the antimicrobial agent of *S. chinensis* spiked to the sample. 

The reported tartaric acid content is *S. chinensis* 8% [25], while the minimum inhibitory concentration (MIC) of tartaric acid is known to be 120.9 ug/mL [26]. Therefore, it is believed that the effective concentration of tartaric acid present in *S. chinensis* is sufficient.

### 2.9. Comparison of UV Sptectum between Tartaric Acid and the Antimicrobial Substance of S. chinensis

Analysis of the antimicrobial agents of *S. chinensis* was performed using NMR, LC-MS, HPLC, and other methods. Tartaric acid was predicted as the active substance. A comparison of UV spectra between tartaric acid and the antimicrobial substance of *S. chinensis* was performed. The results are shown in Figure 6B. Identical wavelength ranges of the UV spectrum were observed between the two materials. Tartaric acid, an organic compound, occurs naturally in many plants, particularly grapes, bananas, and tamarinds. This organic acid is added to other foods to impart a sour taste or as an acidifying agent, such as in vinegar [27], and is commonly used as an antioxidant [28]. Seneviratne et al. [29] reported on the antibacterial effect of organic acids, including tartaric acid, against *S. mutans*. In agreement with the results of a previous study, the results of this study confirmed that tartaric acid was the antimicrobial substance isolated from *S. chinensis*. Therefore, the findings of this study can be used as a reference base for the manufacture of a wide range of products using *S. chinensis*. In the primary purification of *S. chinensis*, considerably high levels of antimicrobial activity were detected by measurement of various organic acids. Instead of additional isolation, primary purification appears adequate when using *S. chinensis* as an antibacterial material. However, in addition to the study of organic acids, further research is warranted to evaluate other active substances.

## 3. Materials and Methods

### 3.1. Materials and Reagents

The Dried *S. chinensis* was purchased from Jindo Herb (Jindo, Korea) and kept at 4 ℃ or room temperature. All reagents, including ethanol (94.5%), n-hexane (95%), ethyl ether (99%), ethyl acetate (99%), chloroform (99.5%), and n-butanol (99%), were purchased from Daejung (Siheung, Republic of Korea).

### 3.2. Experimental Strain and Medium

The bacterial strain *Streptococcus mutans* KCCM 40105 obtained from the Korean Culture Center of Microorganisms (Seoul, Republic of Korea) was used in this experiment. One inoculation loop of the strain was injected into the culture medium of 10 mL Brain Heart Infusion (BHI) broth after growth in a liquid medium, and subculturing was performed three times at 37 ℃. BMI broth was purchased from Difco (Detroit, MI, USA), and agar was purchased from Daejung (Siheung, Republic of Korea).

### 3.3. Measurement of Antimicrobial Activity

After the sample was absorbed onto a paper disc (Advantec 8 mm, Toyo Roshi, Tokyo, Japan), the extraction solvent was completely evaporated under aseptic conditions. The disc was then placed onto the agar plate and incubated at 37 °C for 20–24 h. The antimicrobial activity was determined by measuring the diameter (mm) of the clear zone surrounding the paper disc.

### 3.4. Preparation of S. chinensis s Extract according to Ethanol Concentration

Different concentrations of ethanol by adding 50 mL of solvent adjusted to 0%, 30%, 50%, 70%, and 94.5% ethanol to 5 g of sample, followed by stirring and extraction at room temperature for 24 h. The extracted samples were then filtered through filter paper (Whatman No. 2).

### 3.5. 30% Ethanol Extraction and Solvent Fractionation

A 1 kg sample was extracted in 10 L of 30% ethanol for 24 h, followed by filtering the extract using filter paper (Whatman No. 2) after repeating this process three times. The filtrate was concentrated under reduced pressure on a steam bath at 37 °C using a rotary vacuum evaporator (EYELA, NE-1001, Tokyo, Japan) and freeze-dried (HyperCOOL^TM^, Cooling Trap HC3055, Seoul, Republic of Korea) after removal of water. For solvent fractionation of the sample, 30% ethanol extract powder was suspended in distilled water, and sequential fractionation was performed according to the separation process. The 30% ethanol extract powders of *S. chinensis* and hexane were placed in a separatory funnel at a 1:1 ratio, followed by the separation of the hexane layer. The hexane layer was concentrated under reduced pressure using a rotary vacuum evaporator (EYELA, NE-1001), and the fractions were collected. Using the same procedure, chloroform, ethyl acetate, n-butanol, and water layers were added sequentially to obtain each fraction.

### 3.6. Fractionation by Silica Gel Column Chromatography

**Figure 7:**
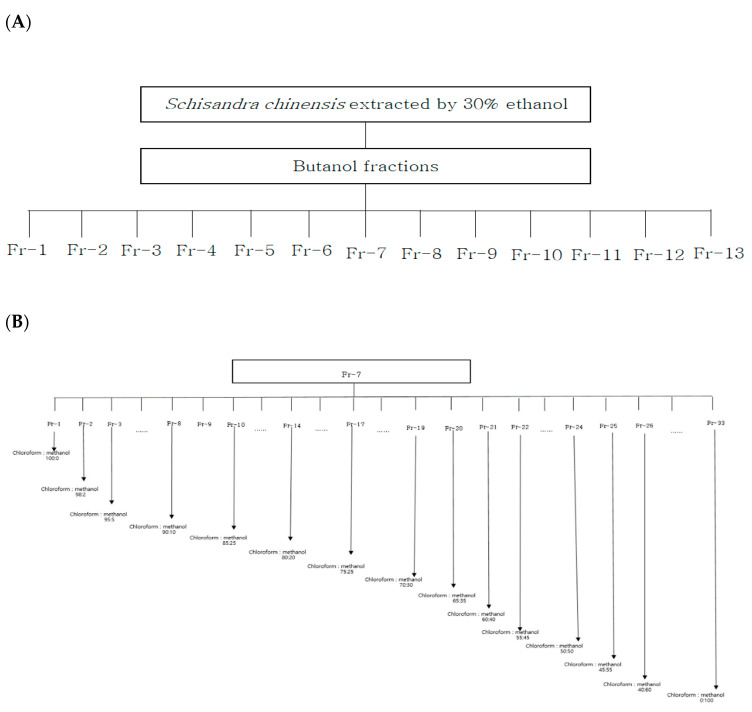
The numbering of *Schizandra chinensis* fractions by silica gel column chromatography (**A**) and the solvent gradient process for 2nd silica gel column chromatography of *Schizandra chinensis* fraction 7 (**B**).

Silica gel column chromatography was performed using silica gel (70–270 mesh, Merck, Darmstadt, Germany) and a glass column (∅25 × 300 mm) for isolation of the active antimicrobial substances from 30% ethanol extract of *S. chinensis*. Then, 10 g of the butanol fraction in powder form was dissolved in methanol and added to a flask for adsorbing onto silica gel. In a glass column packed with silica gel, fractions were separated according to varying polarity using step gradient elution (chloroform 100% → methanol 100%), and 23 fractions were collected. Measurement of absorbance from 210 nm to 1000 nm was performed using thin layer chromatography (TLC) and a microplate reader (SPECTROstar^Nano^, BMG Labtech, Ortenberg, Germany); (Data not shown) 13 fractions including sections showing a similar spectral pattern were concentrated under reduced pressure, and assessment of antimicrobial activities was performed for identification of active sections (Figure 7A). 

For separation by secondary silica gel column chromatography, fractions exhibiting antibacterial activity in primary column chromatography were mixed and allowed to adsorb onto silica gel in a glass column packed with silica gel according to varying polarity using a step gradient-elution system, the solvent from chloroform: methanol (100:1) to chloroform: methanol (1:100). A total of 33 fractions were concentrated under reduced pressure with confirmation of sections exhibiting an active antibacterial effect. These processes are presented in Figure 7B. (① chloroform/methanol = 100:0, ② chloroform/methanol = 98:2, ③ chloroform/methanol = 95:5, ④ chloroform/methanol = 90:10, ⑤ chloroform/methanol = 85:15, ⑥ chloroform/methanol = 80:20, ⑦ chloroform/methanol = 75:25, ⑧ chloroform/methanol = 70:30, ⑨ chloroform/methanol = 65:35, ⑩ chloroform/methanol = 60:40, ⑪ chloroform/methanol = 55:45, ⑫ chloroform/methanol = 50:50, ⑬ chloroform/methanol = 45:55, ⑭ chloroform/methanol = 40:60, ⑮ chloroform/methanol = 35:65, ⑯ chloroform/methanol = 30:70, ⑰ chloroform/methanol = 25:75, ⑱ chloroform/methanol = 20:80, ⑲ chloroform/methanol = 15:85, ⑳ chloroform/methanol = 10:90, ㉑ chloroform/methanol = 5:95, ㉒ chloroform/methanol = 0:100).

### 3.7. Pure Separation by High-Performance Liquid Chromatography (HPLC)

A high level of antimicrobial activity was confirmed in Fraction no. 17 by secondary column chromatography, which was dissolved in methanol, filtered using a 0.45 µm syringe membrane filter, and pure isolation was performed using a µ-Bondapak C_18_ column (4.6 mm × 250 mm, 15–20 µm, waters). HPLC (Waters 1525 and 717, Waters Co., Ltd., Milford, MA, USA) was performed for separation of the fraction in 100% deionized water using gradient elution with 100% acetonitrile; the Waters 996 Detector was used for detection with an injection of 10 µL of the sample.

### 3.8. Analysis of UV Spectrum

Identification was performed in peak no. 2, which exhibited antimicrobial activity in the secondary process of pure isolation using HPLC in antimicrobial substances of *S. chinensis* against the *S. mutans* strain. HPLC was performed for the determination of the UV spectrum in the substance of the second active peak by measuring absorbance in the 210–1000 nm range using a microplate reader (SPECTROstar^Nano^, BMG Labtech).

### 3.9. Molecular Weight Determination by LC-MS

Liquid chromatography–mass spectrometry (LC-MS) (Shimadzu Co., Ltd., Kyoto, Japan) was performed for determination of the molecular weight of the active substance in the second peak of HPLC. The pure active substance in the second peak was isolated by HPLC and injected into LC-MS. Acetonitrile with a flow rate of 0.3 mL/min was used for the mobile phase.

### 3.10. Structure Determination by Nuclear Magnetic Resonance (NMR) Spectroscopy

The molecular structure of the antimicrobial substance in the second peak obtained from *S. chinensis* was determined using NMR spectroscopy. The active substance detected in peak no. 2 in HPLC was filtered using a 0.45 µm membrane filter (Millipore Co., Ltd., Burlington, MA, USA) and dissolved in CDCl_3_ for use. NMR spectra were recorded using a JNM-ECZ 600 (Jeol, Tokyo, Japan) spectrometer. Measurement of ^1^H-NMR and ^13^C-NMR spectra was performed after operating the NMR spectrometer for 30 min for measurement of ^1^H-NMR and 5 hrs for measurement of ^13^C-NMR.

### 3.11. Substance Determination by HPLC

In determining structure using NMR spectroscopy, tartaric acid was predicted as the antimicrobial substance in *S. chinensis*. Therefore, a comparison of chromatograms for the standard of tartaric acid and the antimicrobial substance of *S. chinensis* was performed. HPLC (Waters 1525 and 717, Waters Co., Ltd., Milford, MA, USA) using an organic acid column (ID 4.6 × 250 mm, Grace Co., Ltd., Deerfiled, IL, USA) with a mobile phase of 0.2 mM KH_2_PO_4_ and a flow rate of 1.0 mL/min was performed for comparison of chromatograms. The Waters 996 Detector was used for detection.

### 3.12. Comparison of the UV Spectrum between Tartaric Acid and the Antimicrobial Substance of S. chinensis

In the analysis of antibacterial components of *S. chinensis* using NMR, LC-MS, HPLC, and other methods, tartaric acid was predicted as the active substance. A comparison of the UV spectrum between these two substances was performed. Measurement of absorbance in the range of 210 to 1000 nm was performed using a microplate reader (SPECTROstar^Nano^, BMG Labtech).

### 3.13. Statistical Analysis

All experiments were repeated three times, and analysis of measurements was performed using the SPSS program (26, IBM Corp Armonk, NY, USA). Mean ± SD was calculated, and testing of statistical differences between samples was performed using Duncan’s multiple range test.

## 4. Conclusions

While the antibacterial properties of *Schisandra chinensis* (*S. chinensis*) seeds and fruit extracts have been reported, previous studies have primarily examined their efficacy against foodborne pathogens and commensal skin bacteria. However, there have been few reports on the antibacterial activity against oral bacteria, specifically *Streptococcus mutans (S. mutans)*. Further investigations are required to verify the efficacy against oral bacteria, specifically *S. mutans*. Moreover, the aim was to efficiently eliminate diverse microorganisms that may persist in the oral cavity and establish a foundation for the development of various products with potential antibacterial properties. The aim of this study is to isolate and identify antibacterial agents from *S. chinensis* that exhibit efficacy against the *Streptococcus mutans* KCCM 40105 strain. To achieve the objective, we conducted a series of experiments, including the selection of the appropriate ethanol concentration for *S. chinensis* extraction, identification of suitable solvent fractions, and a stepwise purification process of antibacterial active compounds using silica gel column chromatography and HPLC. The analysis confirmed the identity of the compound as tartaric acid. The findings of this research will provide basic data that can expand the potential uses of *S. chinensis*. This basic data can be utilized in the development of products with the ability to effectively eliminate diverse microorganisms present in the oral cavity and demonstrate antibacterial properties.

## Data Availability

The authors confirm that the data supporting the finding of this study are available within the article.

## Sample Availability

Samples of the compounds of *Schisandra chinensis* are available from the authors.

## References

[1] Jeun E.S., Yoon S.H., Han M.D. (2002). Antimicrobial activity of streptococcus mutans by herbal medicine extracts. J. Dent. Hyg. Sci..

[2] Gálvez A., Abriouel H., López R.L., Ben Omar N. (2007). Bacteriocin-based strategies for food biopreservation. Int. J. Food Microbiol..

[3] Kim M.Y., Hwang H.J., Kang K.H. (2022). Antibacterial and phagocytosis control of natural extracts on *S. mutans*. J. Korea Converg. Soc..

[4] Park E.J., Lee J., Choi W.S. (2011). Anti-cariogenic activities of mushroom extracted with various solvent systems. Korean J. Food Sci. Technol..

[5] Chang G.T., Kang S.K., Kim J.H., Chung K.H., Chang Y.C., Kim C.H. (2005). Inhibitory effect of the korean herbal medicine, dae-jo-whan, on platelet activating factor-induced platelet aggregation. J. Ethnopharm..

[6] Nam S.Y., Lee J.Y., Ko J.S., Kim J.B., Jang H.H., Kim H.R., Lee Y.M. (2014). Changes in antioxidant and antimicrobial activities of *Schizandra chinensis* Baillon under different solvent extraction. J. Korean Soc. Int. Agric..

[7] Choi J.W., Kim J.E., Kim S.Y., Bae Y.S., Kim C.K. (2017). Investigation on the technology trend in omija by the patent index. Korean J. Plant Res..

[8] Kim M.K., Lee J.M., Do J.S., Bang W.S. (2015). Antioxidant activities and quality characteristics of *omija* (*Schizandra chinesis* Baillon) cookies. Food Sci. Biotechnol..

[9] Lee K.S., Lee B.H., Seong B.J., Kim S.I., Han S.H., Kim G.H., Park S.B., Kim H.H., Choi T.Y. (2016). Chemical components composition on different parts of fruit in *Schisandra chinensis* baillon. J. Korean Soc. Food Sci. Nutr..

[10] Xiao W.L., Tian R.R., Pu J.X., Li X., Wu L., Lu Y., Li S.H., Li R.T., Zheng Y.T., Zheng Q.T. (2006). Triterpenoids from *Schisandra lancifolia* with anti-HIV-1 activity. J. Nat. Prod..

[11] Lu Y., Chen D.F. (2009). Analysis of *Schisandra chinensis* and *Schisandra sphenanthera*. J. Chromatogr. A.

[12] Kim H.J. (2014). Isolation of Compounds from the Fruits of *Schisandr chinensis* Baill. Master’s Thesis.

[13] Kim H.J., Seo Y.M., Lee E.J., Chung C.W., Sung H.J., Sohn H.Y., Park J.Y., Kim J.S. (2018). Anti-proliferatice and pro-appoptotic activities by pomace of *Schisandra chinensis* (Turcz.) baill. and Schizandrin. J. Life Sci..

[14] Kim T.I., Choi E.J., Chung C.P., Han S.B., Ku Y. (2002). Antimicorbial effect of *Zea mays* L. and *Magnoliae cortex* extract mixtures on periodontal pathogen and effect on human gingival fibroblast cellular activity. J. Korean Acad. Periodontol..

[15] Jeong P.H., Kim Y.S., Shin D.H. (2006). Changes of physicochemical characteristics of *Schizandra chinensis* during postharvest ripening at various temperatures. Korean J. Food Sci. Technol..

[16] Nam H.H., Kim H.J., Choi N.J., Roh S.S., Choo B.K. (2015). A comparison of antioxidant activity from *Schisandra chinensis* water extracts depending on stir-frying and stir-frying with liquids process. Korean J. Org. Agric..

[17] Jeon E.S., Han M.D., Kim H.D. (2003). Antimicrobial activity of *Streptococcus mutans* by *schizandrae* fructus and *evodiae* fructus extracts. J. Dent. Hyg. Sci..

[18] Kwon H.J., Park C.S. (2008). Biological activities of extracts from *omija* (*Schizandra chinensis* baillon). Korean J. Food Preserv..

[19] Kim J.H., Kim M.J., Choi S.K., Bae S.H., An S.K., Yoon Y.M. (2011). Antioxidant and antimicrobial effects of lemon and eucalyptus essential oils against skin floras. J. Soc. Cosmet. Sci. Korean.

[20] Jung G.T., Ju I.O., Choi J.S., Hong J.S. (2000). The Antioxidative, antimicrobial and nitrite scavenging effects of *Schizandra chinensis* ruprecht(omija) see. Korean J. Food Sci. Technol..

[21] Lee J.Y., Min Y.K., Kim H.Y. (2001). Isolation of antimicrobial substance from *Schizandra chinensis* baillon and antimicrobial effect. Korean J. Food Sci. Technol..

[22] Prosser H.J., Richards C.P., Wilson A.D. (1982). NMR spectroscopy of dental materials. II: The role of tartaric acid in glass-ionomer cements. J. Biomed. Mater. Res..

[23] Ghosh K., Adhikari S. (2006). Fluorescence sensing of tartaric acid: A case of excimer emission caused by hydrogen bond-mediated complexation. Tetrahedron Lett..

[24] Aru V., Sørensen K.M., Khakimov B., Toldam-Andersen T.B., Engelsen S.B. (2018). Cool-climate red wines-chemical composition and comparison of two protocols for ^1^H-NMR analysis. Molecules.

[25] Zhu Y.P. (1998). Chinese Materia Medica: Chemistry, Pharmacology and Applications.

[26] Daglia M., Papetti A., Grisoli P., Aceti C., Dacarro C., Gazzani G. (2007). Antibacterial activity of red and white wine against oral streptococci. J. Agric. Food Chem..

[27] Yi M.R., Kang C.H., Bu H.J. (2017). Acetic acid fermentation properties and antioxidant activity of lemongrass vinegar. Korean J. Food Preserv..

[28] Jeong G.A. (2022). Physicochemical Properties of Tartaric Acid-Treated Korean Wheat Starch and Rice Granule. Master’s Thesis.

[29] Seneviratne C.J., Wong R.W.K., Hägg U., Chen Y., Herath T.D.K., Samaranayake P.L., Kao R. (2011). *Prunus mume* extract exhibits antimicrobial activity against pathogenic oral bacteria. Int. J. Paediatr. Dent..

